# Acrolein induces mtDNA damages, mitochondrial fission and mitophagy in human lung cells

**DOI:** 10.18632/oncotarget.19710

**Published:** 2017-07-31

**Authors:** Hsiang-Tsui Wang, Jing-Heng Lin, Chun-Hsiang Yang, Chun-Hao Haung, Ching-Wen Weng, Anya Maan-Yuh Lin, Yu-Li Lo, Wei-Shen Chen, Moon-Shong Tang

**Affiliations:** ^1^ Department of Pharmacology, National Yang-Ming University, Taipei, Taiwan; ^2^ Faculty of Pharmacy, National Yang-Ming University, Taipei, Taiwan; ^3^ Department of Medical Research, Taipei Veterans, General Hospital, Taipei, Taiwan; ^4^ Department of Environmental Medicine, Pathology and Medicine, New York University School of Medicine, New York, NY, USA

**Keywords:** acrolein, ROS, mtDNA damages, mitochondrial fission, mitophagy

## Abstract

Acrolein (Acr), a highly reactive unsaturated aldehyde, can cause various lung diseases including asthma, chronic obstructive pulmonary disease (COPD), and lung cancer. We have found that Acr can damage not only genomic DNA but also DNA repair proteins causing repair dysfunction and enhancing cells’ mutational susceptibility. While these effects may account for Acr lung carcinogenicity, the mechanisms by which Acr induces lung diseases other than cancer are unclear. In this study, we found that Acr induces damages in mitochondrial DNA (mtDNA), inhibits mitochondrial bioenergetics, and alters mtDNA copy number in human lung epithelial cells and fibroblasts. Furthermore, Acr induces mitochondrial fission which is followed by autophagy/ mitophagy and Acr-induced DNA damages can trigger apoptosis. However, the autophagy/ mitophagy process does not change the level of Acr-induced mtDNA damages and apoptosis. We propose that Acr-induced mtDNA damages trigger loss of mtDNA via mitochondrial fission and mitophagy. These processes and mitochondria dysfunction induced by Acr are causes that lead to lung diseases.

## INTRODUCTION

Acrolein (Acr), a ubiquitous environmental pollutant, is abundant in tobacco smoke, cooking fumes, and automobile exhaust fumes [1–4]. The presence of Acr in the ambient air of urban atmospheres represents a considerable exposure hazard to humans, resulting in various health problems including atherosclerosis and airway diseases such as asthma, chronic obstructive pulmonary disease (COPD), cystic fibrosis, and carcinogenesis [4, 5]. Acr also can be produced endogenously through lipid peroxidation, resulting from free radical damage to polyunsaturated fatty acids [4]. Acr is a strong electrophile and can readily react with nucleophilic reactive groups of biomolecules, including DNA base guanosine yielding mutagenic cyclic propano-deoxyguanosine (PdG) adducts [4]. Our previous studies have shown that Acr-induced PdG adducts induce G to A transitions and G to T transversions [6–10]. In addition, Acr also reacts with cysteine, histidine and lysine residues of proteins, resulting in inactivation of proteins [4]. We also found that Acr impairs DNA repair function through modifications with repair proteins and further enhances cells’ susceptibility to DNA damage-induced mutagenesis [6–9].

Mitochondria have an essential function in all cells, providing cellular energy by generating ATP via respiration using several crucial mitochondrial DNA (mtDNA)-encoded proteins. As such, maintaining the integrity of mtDNA is essential for healthy life [11, 12]. Due to the lack of protective histones and introns, mtDNA is susceptible to damage by environmental carcinogens, as well as by endogenous reactive oxygen species (ROS), byproducts of the oxidative phosphorylation system (OXPHOS) [13]. It has been found that the frequency of somatic mtDNA mutations is approximately 10 times higher than that of nuclear genomic DNA (nDNA) [13–15]. Mutations in mtDNA, such as large-scale deletions, D-loop mutations and copy number alterations, have been established as responsible for, or associated with, aging and common diseases of the elderly, including cancer, diabetes, and neurodegenerative disease [14–18].

Mitochondria are dynamic organelles; mtDNA is constantly undergoing fusion and fission, the mitophagy process, during cell life [19]. Removing damaged mtDNA or mitochondria plays an important role in preserving the integrity of the mitochondrial genome [11, 12, 20]. Mitophagy, a selective autophagy pathway, is an evolutionarily conserved homeostatic process by which the cells electively degrade only damaged mitochondria [21]. It has been suggested that mitochondrial fission precedes mitophagy [22]. Recent studies suggested that E3 ubiquitin ligase, Parkin/PARK2 and phosphatase and tensin homolog (PTEN)-induced putative protein kinase1 (PINK1) act as master regulators in the elimination of abnormal mitochondria [23–25]. Thus, mitophagy prevents healthy cellular networks from mitochondrial dysfunction by sequestering the damaged mitochondria. When this fails, mitophagy acts as a prelude to cell death. Failure in the balance of these processes triggers mitochondrial dysfunction, and may lead to cell death.

In this study, we show that Acr induces many facets of mitochondrial injuries such as mtDNA damages, as well as reductions of mtDNA copy number, mitochondrial RNA (mtRNA) transcripts, cellular ATP levels, mitochondrial membrane potential and mitochondrial respiration. However, we found that autophagy/mitophagy induced by Acr does not contribute to cytotoxicity. These results suggest that Acr induces lung diseases other than lung cancer via induction of mitochondrial dysfunction and reduction of mtDNA copy number.

## RESULTS

### Apoptosis is a major pathway of Acr-induced cytotoxicity in human lung epithelial A549 cells and human lung MRC-5 fibroblasts

**Figure 1:**
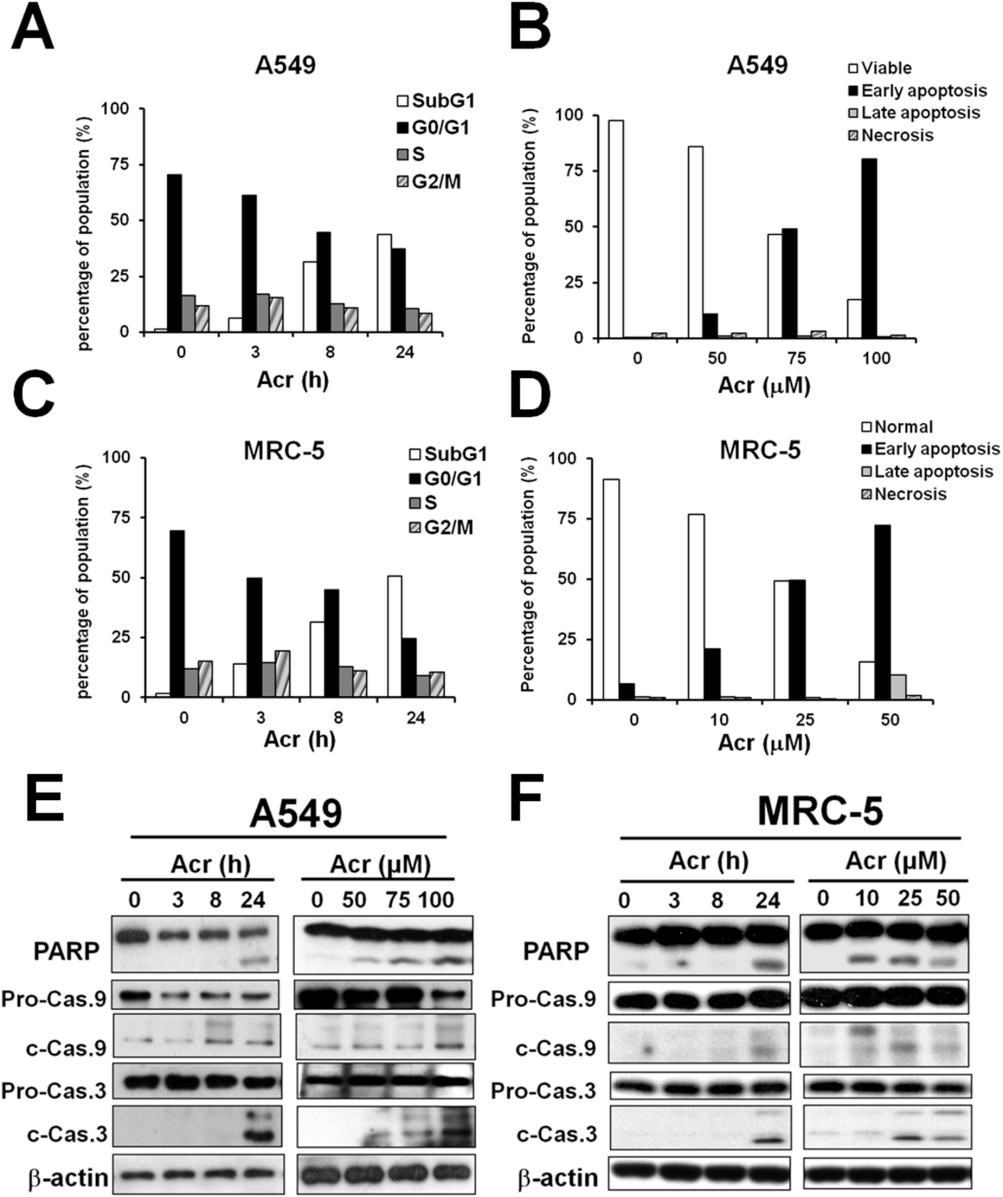
Acrolein-induced cytotoxicity occurs primarily via apoptosis Cells were treated with Acr (A549: 75 μM; MRC-5: 25 μM) for different times (0-24 h) or treated with different concentrations of Acr (A549: 0-100 μM; MRC-5: 0-50 μM), then incubated for 24 h. Panels **(A & C)** show cells in different cell cycle phases as determined by propidium iodide (PI) staining and flow cytometry analysis. Panels **(B & D)** show Acr induced apoptosis and necrosis as analyzed by Annexin V and PI staining and flow cytometry analysis. Panels **(E & F)** show Acr-induced cleavage of PARP, caspase 9 and caspase 3 in Acr-treated A549 or MRC-5 cells in Western blot analyses.

Since aerodigestive organs are the major targets of Acr, and Acr induces asthma, COPD, and lung cancer, we used human lung cells including the epithelial adenoacarcinoma A549 cells and MRC-5 normal lung fibroblasts to dissect the mechanisms underlying Acr-induced cytotoxicity. Results in Figure 1 show that Acr treatment markedly increased the sub-G1 phase (Figure 1A & 1C, Supplementary Figure 1), and the early apoptotic (Figure 1B & 1D, Supplementary Figure 2) populations in both cell lines in time- and dose-dependent manners. Accordingly, increased cleavages of caspase-9 and -3, as well as cleavage of PARP, were observed in both cells treated with Acr (Figure 1E & 1F). Together, these results indicate that Acr induces apoptosis in lung epithelial cells and fibroblasts.

### Acrolein treatment reduces mitochondrial membrane potential, ATP content, and suppresses mitochondrial respiration

**Figure 2:**
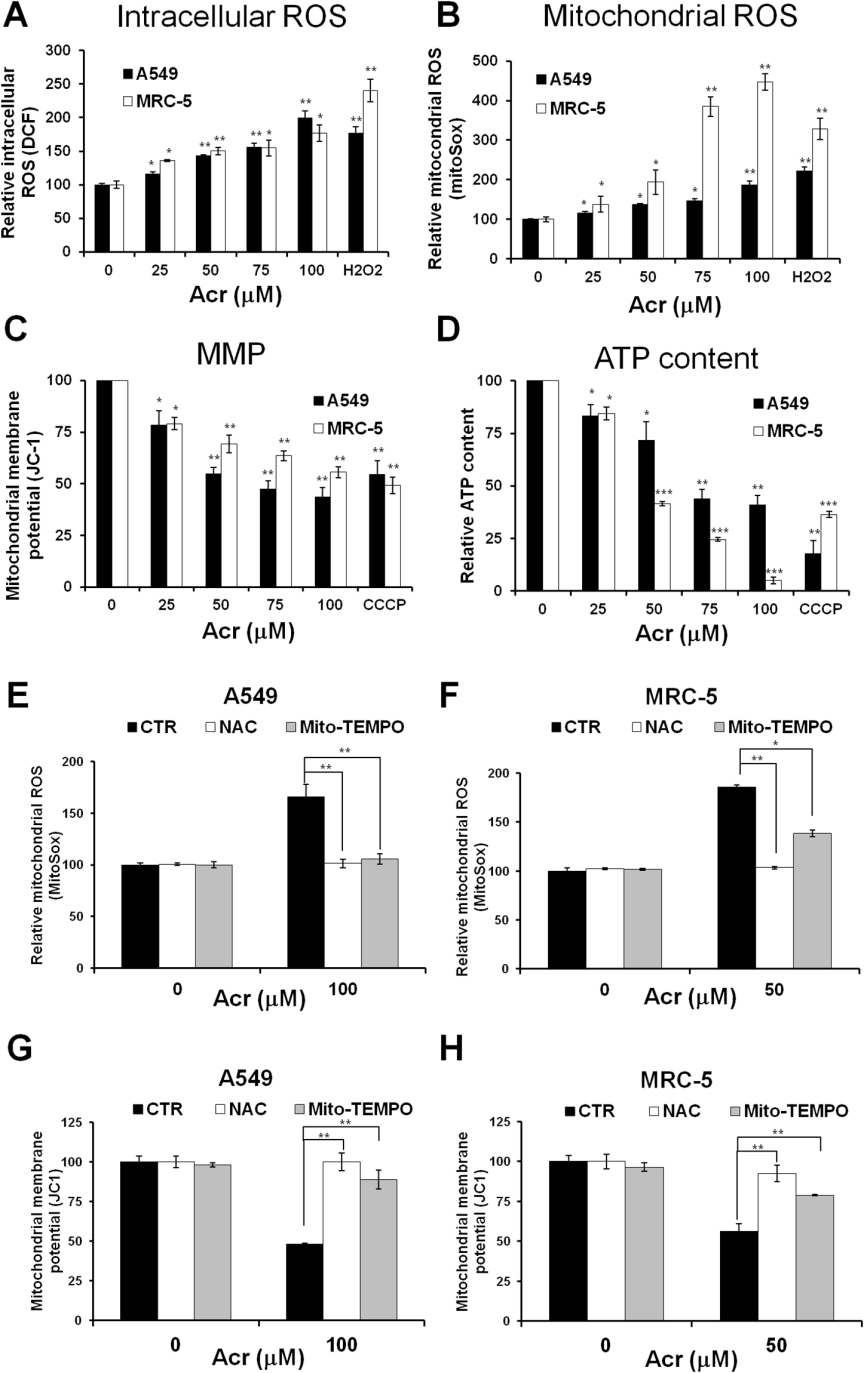
Acrolein induces mitochondrial ROS over-production, mitochondrial membrane potential and ATP content reduction, and NAC or Mito-TEMPO pretreatment eliminates these effects In panels **(A & B)**, cells were treated with different concentrations of Acr (0-100 μM) or H_2_O_2_ (250 μM) for 1 h, and (A) the intracellular and (B) mitochondrial ROS production were detected by DCF and MitoSOX Red staining assays, respectively. In panels **(C & D)**, cells were treated with different concentrations of Acr (0-100 μM) or CCCP (100 μM) for 3 h and (C) the mitochondrial membrane potential (MMP) and (D) the ATP content were measured by JC-1 assay for MMP and ATP bioluminescence assay kits, respectively. In panels **(E-H)**, cells were pretreated with NAC (5 mM) or Mito-TEMPO (100 μM) for 1 h and then subjected to Acr treatment (A549: 100 μM; MRC-5: 50 μM) for 1 h; the (E & F) mitochondrial ROS production and the (G & H) MMP were measured as described above. Bar graphs show data collected from 3 independent experiments. Data are mean ± s.d. * P< 0.05; ** P<0.01; ***P<0.005.

**Figure 3:**
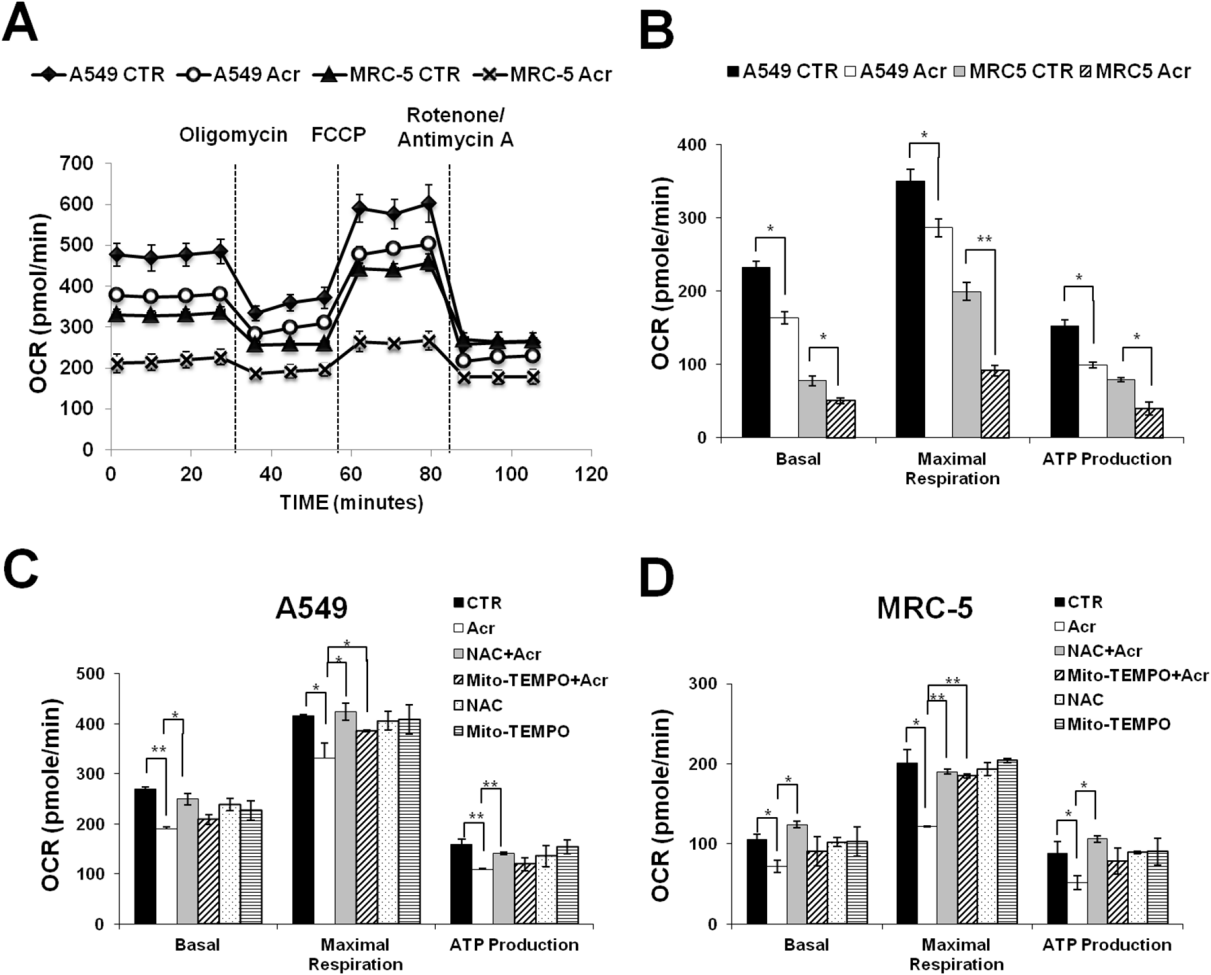
Acrolein inhibits mitochondrial respiration Cells were treated with Acr (A549: 100 μM; MRC-5: 50 μM) for 1 h. The oxygen consumption rate (OCR) was determined using Seahorse XFe24 Metabolic Flux Analyzer. In panel **(A)**, group data of original tracing show changes in OCR in response to the sequential administration of oligomycin (1 μM), carbonyl cyanide 4-(trifluoromethoxy)pheyl- hydrazone (FCCP; 0.25 μM), and rotenone/ antimycin A (1μM). In panel **(B)**, bar graphs are of data collected from 10 independent experiments. Note: Acr exposure decreases basal respiratory, maximal respiratory capacity, and ATP production of mitochondrial in A549 and MRC-5 cells. Data are mean ± s.d. * P< 0.05; ** P<0.01. In panels **(C & D)**, cells were pretreated with NAC (5 mM) or Mito-TEMPO (100 μM) for 1 h then, subjected to the Acr treatment (A549: 100 μM, MRC-5: 50 μM), and the mitochondrial respiration was analyzed, as described above. Bar graphs are of data collected from 10 independent experiments. Note: Pretreatment of NAC or Mito-TEMPO reversed decreased basal respiratory, maximal respiratory capacity, and ATP production of mitochondrial in Acr-treated A549 and MRC-5 cells. Data are mean ± s.d. * P< 0.05; ** P<0.01.

Acr is abundant in tobacco smoke [1, 4] and Acr is a mitochondrial toxin [26]. It is likely that mitochondrial dysfunction caused by Acr may play an important role in its toxicity in smoking-related diseases [27–29]. To test this possibility, we determined the effect of Acr on mitochondria function and integrity. Results in Figure 2 show that Acr increases production of intracellular and mitochondrial ROS (mtROS) (Figure 2A & 2B) and causes dose-dependent decreases of mitochondrial membrane potential (MMP) (Figure 2C) and ATP content (Figure 2D). Pretreatment of cells with an antioxidant, N-acetylcysteine (NAC) or a mitochondria specific antioxidant, Mito-TEMPO rescued Acr-induced ROS production from mitochondria (Figure 2E & 2F), MMP reduction (Figure 2G & 2H) and cytotoxicity (Supplementary Figure 3). To further analyze the effects of Acr on mitochondrial respiration, we measured the oxygen consumption rate (OCR) in A549 and MRC-5 cells using Seahorse Bioscience’s XF96 Analyzer. Results in Figure 3 show that significant reductions in basal respiration, ATP turnover, and maximal respiratory capacity were observed in A549 and MRC-5 cells after a short (1 h) exposure with Acr (Figure 3A & 3B), and show that NAC or Mito-TEMPO pretreatment reverted Acr-induced decreases in basal respiration, ATP turnover, and maximal respiratory capacity of the mitochondria to normal levels (Figure 3C & 3D). Collectively, these results suggest that Acr suppresses mitochondrial respiration via overproduction of mtROS.

### Acrolein induces bulky DNA damages in mitochondria and alteration of mtDNA copy number

**Figure 4:**
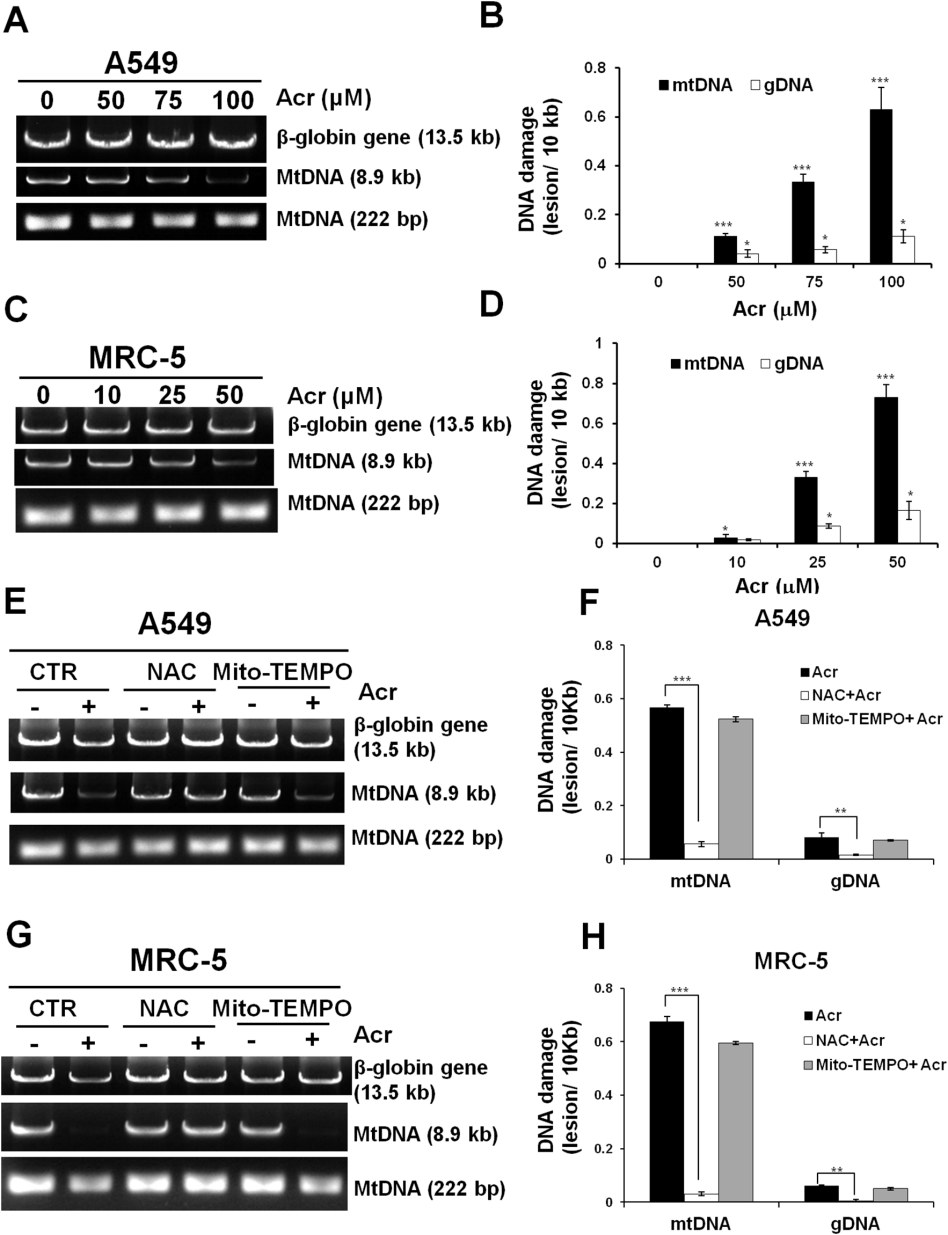
Acrolein induces more DNA damages/ unit length DNA in mtDNA than in nDNA In panels **(A & C)**, cells were treated with Acr (A549: 0-100 μM, MRC-5: 0-50 μM for 6 h), DNA was extracted, following by QPCR with specific primers against mtDNA (8.9kb, 222bp) and nDNA (β-globin, 13.5kb). The resultant DNAs were separated by electrophoresis in a 1% agarose gel as previously described [67]. Panels **(B & D)** show quantifications of DNA damages in mtDNA and nDNA. In panels **(E & G)**, cells were pretreated with NAC (5 mM) or Mito-TEMPO (100 μM) for 1 h followed by Acr treatment (A549: 100 μM, MRC-5: 50 μM) for 6 h. DNA damages in mtDNA and nDNA damages were analyzed, the same as described above. Panels **(F & H)** show quantifications of Acr-induced DNA damages in mtDNA and nDNA in cells pretreated with NAC (5 mM) or Mito-TEMPO (100 μM). Bar graphs show data collected from 3 independent experiments. Data are mean ± s.d. * P< 0.05; ** P<0.01; ***P<0.005.

The aforementioned results show that Acr causes a variety of mitochondrial toxicities and raise the possibility that Acr is able to penetrate mitochondrial membranes to induce mtDNA damages. To test this possibility, we directly determined mtDNA damages in Acr-treated cells. Using a well-validated, sensitive QPCR assay that relies on the principle that DNA lesions inhibit DNA polymerases (e.g., *Taq* and *rTth* DNA polymerase) as previously described [30], we found that more mtDNA damages occurred compared to nDNA damages in A549 and MRC-5 cells treated with Acr (Figure 4A-4D). These results are consistent with previous results showing that mtDNA is more susceptible than nDNA to endogenous or exogenous DNA damage agents [13–15]. Pretreatment with NAC, but not Mito-TEMPO, reduced the observed mtDNA damages induced by Acr (Figure 4E-4H).

Further, we have established that Acr induces cyclic α- and γ-hydroxy- propano-dG adducts (PdG) in genomic DNA in lung epithelial cells and fibroblasts and that Acr-induced PdG adducts are sensitive to nucleotide excision repair (NER) enzyme UvrABC incision [31, 32]. Using the UvrABC incision assay in combination with Southern blot hybridization [33] allowed us to determine whether or not Acr induces PdG adducts in mtDNA. The results in Supplementary Figure 4A & 4B show that Acr induced dose-dependent UvrABC sensitive sites in mtDNA, indicating that Acr induces bulky PdG adducts. Using T4 endonuclease V (T4 endo V) incision and density centrifugation, Clayton, *et al*, have reported that mitochondria lack NER [20]. However, the method used in this prior work requires isolation of covalently closed circle mtDNA which may have excluded the repaired and repairing mtDNA. To confirm the lack of NER in mtDNA, we determined the repair of UV-induced cyclobutane pyrimidine dimers (CPDs) directly using T4 endonuclease V incision followed by Southern blot hybridization, visualizing all mtDNA including open circle repair intermediates and closed circle mtDNA. The results in Supplementary Figure 4C & 4D show that indeed mtDNA are unable to repair CPDs. Consistently, we found that Acr-induced mtDNA damages cannot be repaired using QPCR assay (Supplementary Figure 4E & 4F). Together, these results indicate that Acr induces bulky PdG adducts in mtDNA and that mitochondria are unable to repair these DNA adducts.

**Figure 5:**
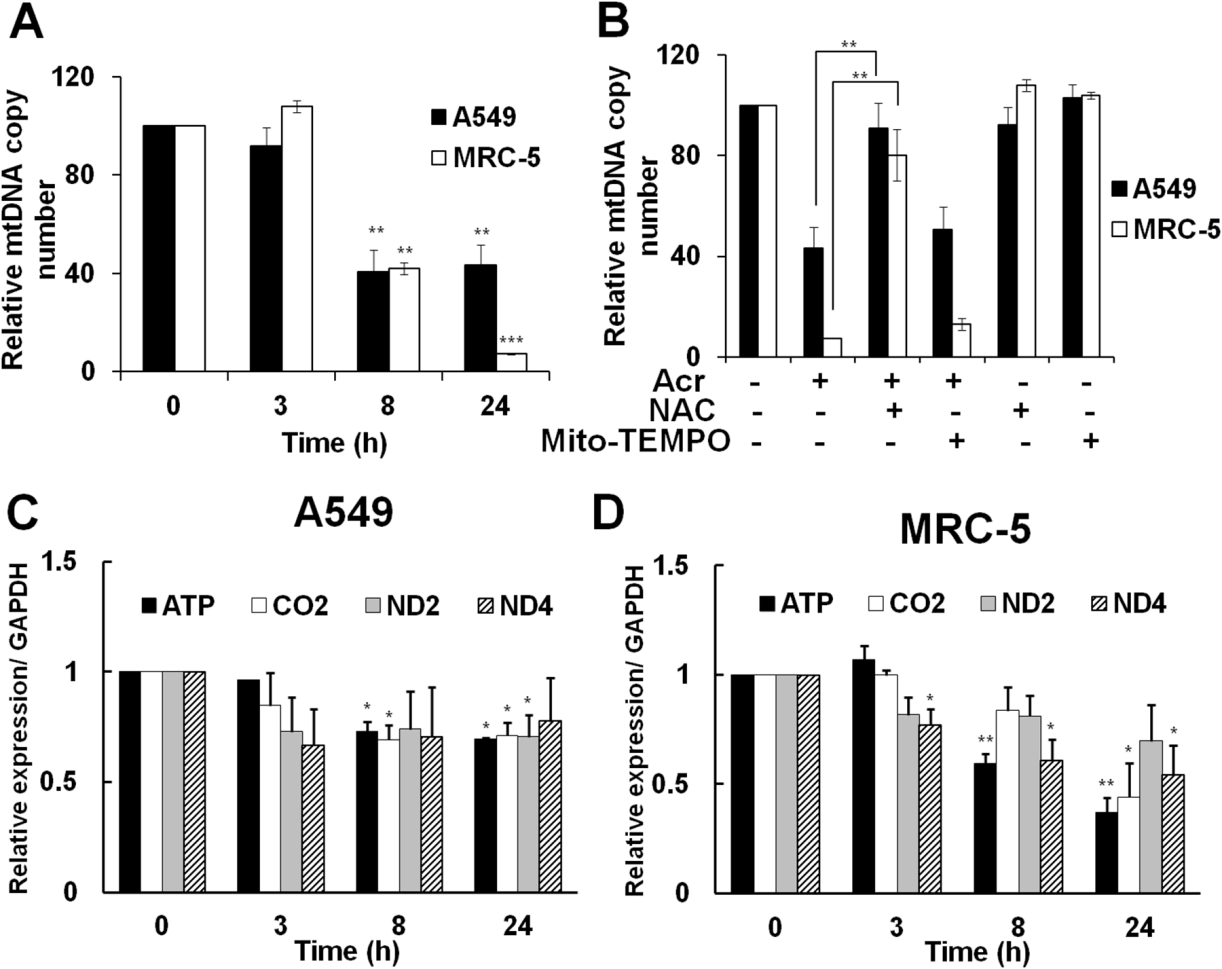
Acrolein alters mtDNA copy number and reduces expression of mitochondria specific genes and NAC can neutralize these effects In panel **(A)**, cells were treated with Acr (A549 75 μM; MRC-5: 25 μM) for different times (0-24 h) and the relative mtDNA copy number was analyzed by quantitative real-time PCR with specific primer against D-region of mtDNA and nuclear 18S gene, as described in materials and methods. In panel **(B)**, effect of NAC or Mito-TEMPO on mtDNA copy number in Acr-treated A549 and MRC-5 cells. Cells were pre-treated with NAC (5 mM) or Mito-TEMPO (100 μM) for 1 h, and then treated with Acr (A549: 75 μM; MRC-5: 25 μM) for 24 h. The mtDNA copy number was determined, as described above. In panels **(C & D)**, mitochondria specific genes (ATP, CO2, ND2, and ND4) were analyzed in Acr-treated A549 and MRC-5 cells. After cells were treated with Acr, as described in panel A, RNA was extracted, followed by RT-PCR with specific primers against mitochondrial genes. Bar graphs show data collected from 3 independent experiments. Data are mean ± s.d. * P< 0.05; ** P<0.01.

Since the maintenance of mtDNA quality is crucial for mitochondrial homeostasis, accumulation of damaged mtDNA may induce mutated mtDNA, including mtDNA copy number alterations [12]. Results in Figure 5 show that Acr reduced mtDNA copy number in both A549 and MRC-5 cells (Figure 5A) and that pre-treatment of NAC, but not Mito-TEMPO, reversed the reduction of mtDNA copy number (Figure 5B). In addition, we found that Acr also reduced expression of mitochondrial genes (Figure 5C & 5D) parallel to the reduction of mtDNA copy number. These results indicate that Acr-induced mtDNA damages result in alteration of mtDNA copy number, and reduction of mitochondrial gene expressions.

### Acrolein induces mitochondrial fission followed by mitophagy

**Figure 6:**
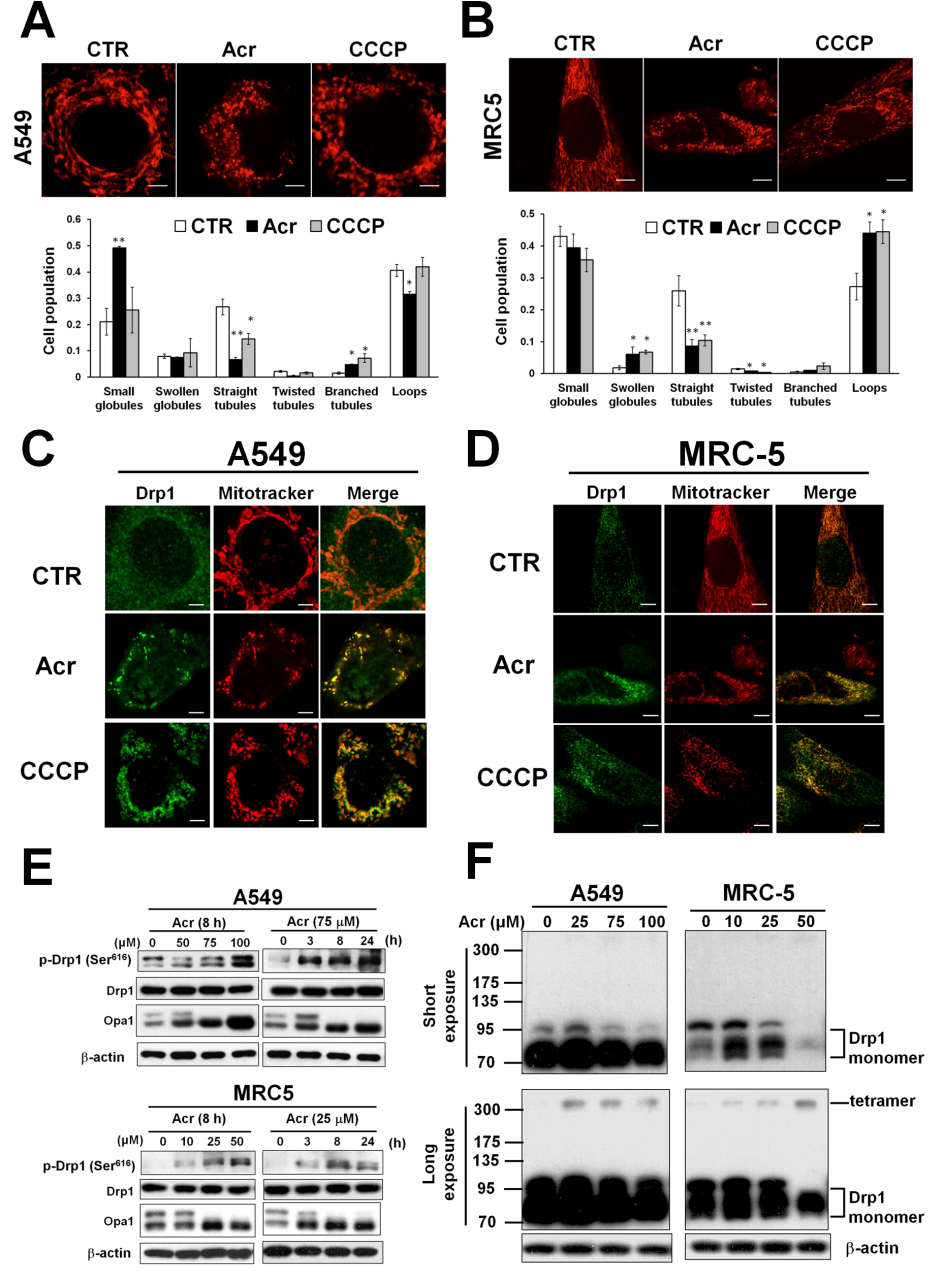
Acrolein induces mitochondrial fission In panel **(A & B)**, A549 and MRC-5 cells were treated with Acr (A549: 75 μM, MRC-5: 25 μM) for 8 h, and stained with mitotracker (upper) and the mitochondrial morphology change was quantified (lower). Bar graphs show data collected from 3 independent experiments. Data are mean ± s.d. * P< 0.05; ** P<0.01. In panels **(C & D)**, Drp1 translocation was detected by immunofluorescent staining assay. A549 and MRC-5 cells were treated with Acr (A549: 75 μM, MRC-5: 25 μM) or CCCP (20 μM) for 8 h at 37°C, fixed, stained with Drp1, and then examined by microscopy. Mitotracker was used to stain mitochondria. Phosphorylated Drp1 (p-Drp1, Ser616), Drp1, OPA1 (panel **E**) and tetramerization of Drp1 (panel **F**) in Acr-treated cells was detected by Western blot analysis. Note: Acr induces phosphorylation of Drp1 at Ser616 site followed by tetrameriaztion of Drp1 and cleavage of OPA1 indicating that Acr induces mitochondrial fission.

Mitophagy is a mitochondrial maintenance pathway that helps preserve the integrity of the mitochondrial genome [21]. It has been suggested that mitochondrial fission precedes mitophagy in order to remove damaged mitochondria [22]. We found that A549 and MRC-5 cells treated with Acr had mitochondria with a fragmented, punctiform morphology unlike control cells that exhibited normal, short, tubular mitochondria (Figure 6A & 6B). Phosphorylation of dynamin-related protein (Drp1) at serine 616 is a post-translational modification known to cause Drp1 to translocate to the mitochondria forming multimers that circumferentially constrict the mitochondrion and initiate fission [34]. To clarify the molecular mechanism of mitochondria fission in response to Acr exposure, we performed immunofluorescent staining assay to determine the co-localization of fission protein (Drp1) with mitotracker (Figure 6C & 6D). We found that Acr did not change total Drp1 expression. However, it did increase expression of activated Drp1 (Drp1-p-Ser-616) following by tetramerization of Drp1 (Figure 6E & 6F). On the other hand, the dynamin-like GTPase OPA1 functions in mitochondrial fusion and inner membrane remodeling and it has been suggested that proteolytic cleavage of long form OPA1 to the short form limits fusion and can facilitate mitochondrial fission [35–37]. Consistently, cleavage of fusion protein OPA1 to short form was observed in Acr-treated cells (Figure 6E). These data demonstrate that Acr induces mitochondrial fission.

**Figure 7:**
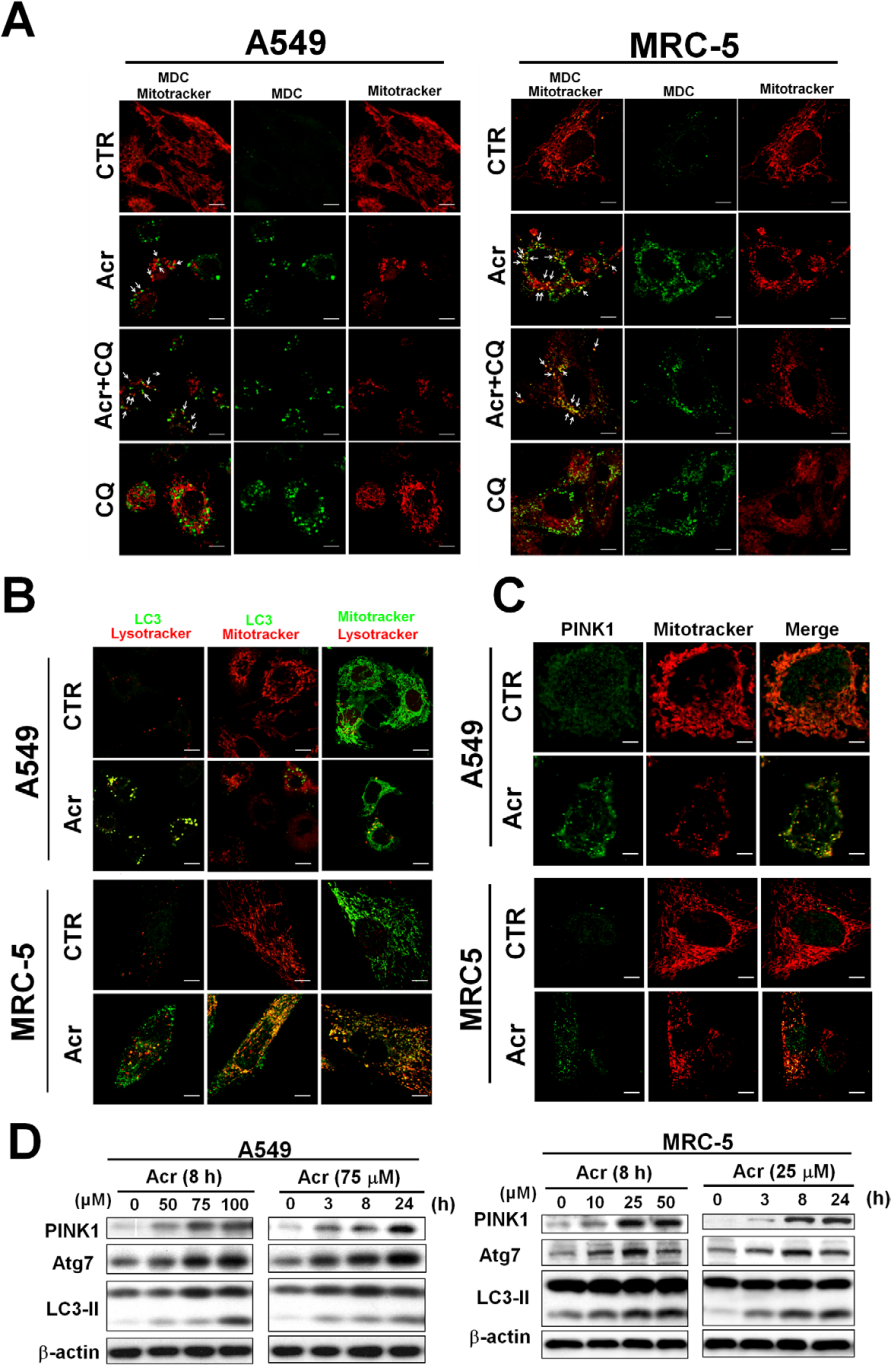
Acrolein induces mitophagy in lung cells In panel **(A)**, A549 and MRC-5 cells were treated with Acr (A549: 75 μM; MRC-5: 25 μM) and/ or chloroquine (CQ, 20 μM) for 8 h at 37°C, and autophagosome were detected by the monodansylcadaverine (MDC) immunofluorescent staining methods [63]. Note: MDC staining and mitotracker were used to detect the autophagic vacuoles and mitochondria. Because MDC accumulates in mature autophagic vacuoles, such as autophagosomes but not in the early endosomal compartment, MDC staining can be used to detect autophagic vacuoles. In panels **(B & C)**, LC3-II and translocation of PINK1 were detected by immunofluorescent staining assay. Cells were treated with Acr (A549: 75 μM; MRC-5: 25 μM for 8 h), fixed, stained with PINK1 or LC3 antibody, followed by goat anti-rabbit FITC -conjugated secondary antibody, then examined by microscopy. Mitotracker and Lysotracker were used to stain mitochondria and lysosome, respectively. In panel **(D)**, Western blot analysis of PINK1, Atg7, and cleavage of LC3 in A549 and MRC-5 cells treated with different concentrations and times of Acr, as indicated.

Using MDC staining, the bright green dots, which indicate autophagosomes, increased in the cytoplasm of Acr-treated A549 and MRC-5 cells and co-localized with mitochondria stained with Mitotracker Red as indicated by arrows (Figure 7A). Further, LC3 punta were formed in Acr-treated cells and superimposed with mitochondria by immunofluorescent staining assay (Figure 7B). Consistently, we found that Acr treatment in A549 and MRC-5 cells increased Atg7 and the formation of LC3-II, a hallmark of the autophagy pathway (Figure 7D). In addition, PINK stabilization and translocation to mitochondria were detected by Western blot and immunofluorescent staining analyses, respectively (Figure 7C & 7D). Colocalization of lysosomes stained by lysotracker with mitochondria stained by Mitotracker Red or with LC3 punta by immunofluorescent staining assays (Figure 7B) was also observed in cells treated with Acr. Together, these data indicate that Acr induces the mitophagy pathway which is followed by mitochondrial fission.

### Mitophagy does not restore Acr-induced mtDNA damages and cytotoxicity

**Figure 8:**
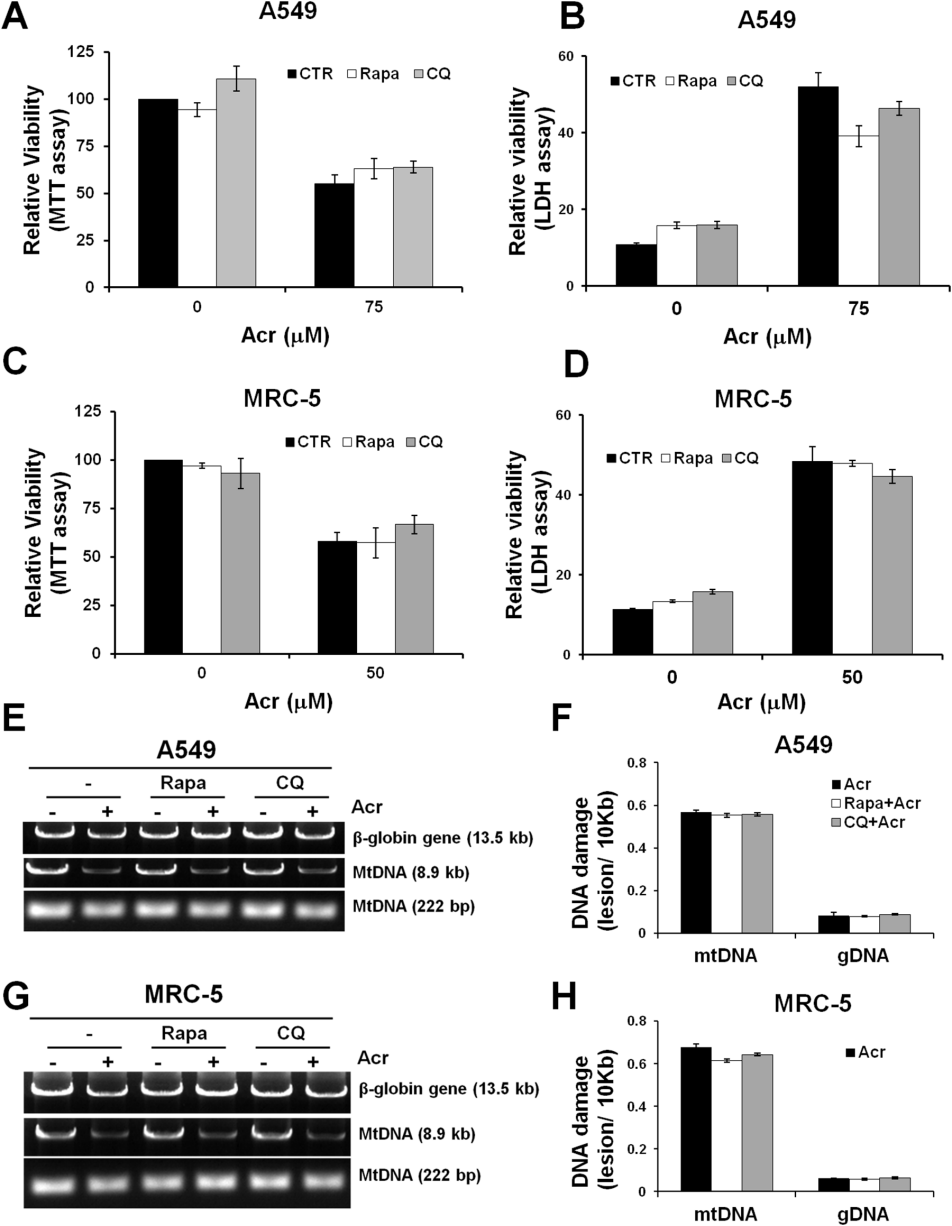
Mitophagy/ autophagy does not restore acrolein-induced cytotoxicity and DNA damages A549 or MRC-5 cells were pretreated an autophagy inducer, rapamycin (Rapa,100 μM) or an autophagy inhibitor, chloroquine (CQ, 20 μM) for 1 h, then co-treated with Acr (A549: 75 μM; MRC-5: 25 μM) for 24 h at 37 °C. Relative viability was determined by (panels **A & C**) MTT assay and (panels **B & D**) LDH leakage assay. Panels **(E-H)** show the effects of autophagy inhibitors or inducers on Acr-induced DNA damages. A549 or MRC-5 cells were pretreated rapamycin (Rapa, 100 μM) or chloroquine (CQ, 20 μM) for 1 h, then co-treated with Acr (A549: 75 μM; MRC-5: 25 μM) for 6 h at 37 °C, and mtDNA and nDNA damages were analyzed (panels E & G), as described in Figure 4, and quantified as shown (panels F & H). Bar graphs show data collected from 3 independent experiments. Data are mean ± s.d.

Since Acr treatment induces autophagy/mitophagy we then determined the effect of these processes on cytotoxicity. Using the MTT assay and LDH leakage assays, we found that pre-treatment of cells with either an autophagy inducer, rapamycin, or an autophagy inhibitor, chloroquine (CQ) did not significantly reduce Acr-induced cell death (Figure 8A-8D). This result indicates that autophagy/ mitophagy does not have an effect on Acr-induced cytotoxicity. Since the function of the mitophagy pathway is to remove damaged mitochondria or mtDNA, we analyzed Acr-induced mtDNA damages after pre-treatment of rapamycin or CQ. The results show that Acr-induced mtDNA damages did not change significantly after rapamycin or CQ pre-treatment (Figure 8E & 8F, Supplementary Figure 5). Furthermore, LC3 is essential for fusion of autophagosome with its target membrane during autophagy/ mitophagy pathway [38]. In order to confirm the above results, we used LC3 shRNA plasmid transfection for inhibition of expression of LC3 and found consistent results (Supplementary Figure 6). This indicates that mitophagy does not enhance removal of Acr-induced mtDNA damages. These results raise the possibility that Acr-induced release of mitochondrial caspases and mtDNA damage mediate cellular apoptosis (Figure 1).

## DISCUSSION

We found that Acr induces more DNA damages in mtDNA than in nDNA (Figure 4). There are two probable mechanisms that can account for these results. One, the location of mitochondria is more accessible than the nucleus for exogenous Acr to induce DNA damages; and two, mitochondria lack nucleotide excision repair (NER) mechanisms. Although base excision repair enzymes are found in mitochondria, it has previously been reported that mitochondria are deficient in NER processes [20]. In this present study, we confirmed that mtDNA are deficient in NER (Supplementary Figure 4C), and that they are much more vulnerable to Acr-induced damages than nDNA (Figure 4). Our results show that 25 μM of Acr (24-72 hr) induced 4977 deletions, the most common type of mutation found in mtDNA [39] (Supplementary Figure 7), and also resulted in the depletion of mtDNA copy number (Figure 5A). Previous studies have shown that mtDNA is more susceptible to damage than nDNA, in fact the frequency of somatic mtDNA mutations is approximately 10 times higher than that for nDNA [13–15]. Our results provide a plausible explanation.

The mechanisms of Acr-induced cytotoxicity have been suggested to be linked to oxidative stress [40] and mitochondrial dysfunction [26–29]. Overproduction of mitochondrial ROS (mtROS) can induce mitochondrial dysfunction [41]. Mitochondrial dysfunction-elicited ROS production axis forms a vicious cycle [42]. Acr has been shown as a direct oxidant [40], but also as a generator of oxidants by different pathways due to the fast depletion of cellular glutathione (GSH) by Acr [43–46]. In the present study, a GSH pro-drug, NAC or a mitochondrial targeted antioxidant, Mito-TEMPO decreased Acr-induced mtROS and restored mitochondrial bioenergetics (Figures 2E-2H, 3C & 3D) suggesting that Acr-induced mtROS inhibits mitochondrial respiration. Interestingly, the protective effect of NAC is better than Mito-TEMPO against Acr-induced mtROS and mitochondrial dysfunction (Figures 2E-2H, 3C & 3D). Mito-TEMPO did not significantly change Acr-induced mtDNA damages (Figures 4E-4G) or copy number reduction (Figure 5B), which is possibly due to the presence of bulky PdG adducts in mtDNA (Supplementary Figure 4A). Since Acr is highly reacted with sulfhydryl group-containing molecules [4], these results suggest that NAC acts not only as an anti-oxidant, but it also acts as a scavenger of Acr.

Our previous results showed that small airway epithelial (SAE) cells containing mtDNA are more sensitive to Acr-induced cytotoxicity compared with SAE cells without mtDNA (Rho depleted) [7], which indicates that Acr-induced mtDNA damages is involved in Acr-induced cytotoxicity. Given the fact that extensive mtDNA damage or depletion triggers apoptosis, our results lend credit to the hypothesis that in addition to oxidative stress, Acr induces cytotoxicity through mtDNA damages which leads to mitochondrial apoptosis.

Mitochondrial dynamics, including the processes of fission and fusion, allow the intermixing of metabolites and mtDNA, the proliferation and distribution of mitochondria, and cellular adaptation to energy demands [47]. Mitochondrial fission not only facilitates the appropriate distribution of mitochondria according to the local energy demand, it also helps the removal of damaged mitochondria through mitochondrial-specific autophagic degradation (mitophagy) [48–50]. Mitochondrial dynamics are regulated by the balance of expression levels between mitochondrial fission (Fis1 and Drp1) and fusion (MFN1, MFN2, and OPA1) proteins [47, 51, 52]. Posttranslational modification of Drp1 is a prerequisite for translocation to mitochondria, which is a fundamental process during mitochondrial fission [53]. Here, we found that Acr induces phosphorylation of Drp1 and tetramerization of Drp1 results in Drp1 translocation to mitochondria (Figure 6C & 6D). The regulation of mitochondrial dynamics by the GTPase OPA1, which is located at the inner mitochondrial membrane, is crucial to adapting mitochondrial function and preserving cellular health [35–37]. OPA1 governs the delicate balance between fusion and fission in the dynamic mitochondrial network. Long, membrane-bound forms of OPA1 are required for mitochondrial fusion, but their processing to short, soluble forms limits fusion and can facilitate mitochondrial fission. Previous studies have shown that dissipation of the mitochondrial inner membrane potential induces mitochondrial fission by inhibiting OPA1-dependent mitochondrial fusion or activating Drp1-dependent mitochondrial fission [35, 54]. Consistently, we found that Acr causes mitochondrial fission and that the mechanisms of this process involve the activation of Drp1 and cleavage of OPA1 (Figure 6).

Fragmented damaged mitochondria are generally destined to be eliminated by mitophagy [48, 55]. Mitophagy, an evolutionarily conserved homeostatic process, is essential for maintenance of mtDNA integrity [21]. Here, we found that Acr induces the mitophagy pathway, however, inhibition or induction of the mitophagy/ autophagy pathway does not restore Acr-induced mtDNA damages (Figure 8E-8G, Supplementary Figure 6). This indicates that mitophagy fails to remove Acr-induced mtDNA damages. Therefore, it is possible that Acr not only induces the disruption of mitochondrial dynamics but also causes impairment of mitochondrial degradation by mitophagy, resulting in accumulation of fragmented mitochondria (Figure 6A & 6B).

Previous studies demonstrated that excessive mitochondrial fission facilitates apoptosis [56]. We found that Acr induces the mitochondrial apoptosis pathway including cleavages of caspase 9 and caspase 3 (Figure 1). Acr has been shown to trigger either apoptotic or necrotic pathways, depending on the cell types, culture conditions, or even medium composition used [4]. For example, Acr has been demonstrated to induce apoptosis either through intrinsic pathways or extrinsic pathways [57, 58]. Our previous studies have shown that Acr induces ribosomal stress which leads to activation of MDM2 and RPL11-MDM2 binding, consequently, activating p53 and enhancing E2F-1 degradation, and that taken together these two processes induce apoptosis [59]. Here, we found that Acr-induced mitophagy pathways fail to remove Acr-induced mitochondrial damages, and that in turn, these damaged mitochondria induce the mitochondrial apoptosis pathway.

## MATERIALS AND METHODS

### Cell culture and chemical treatment

Normal human lung fibroblasts (MRC-5) and lung adenocarcinoma cells (A549) were grown in minimum essential medium (MEM) supplemented with 10% FBS and RPMI 1640 medium supplemented with 10% FBS, respectively. Cells at 50% confluency were used for Acr treatment as previously described [59]. All chemicals including Acr, H_2_O_2_, carbonyl cyanide 3-chlorophenylhydrazone (CCCP), chloroquine (CQ), rapamycin, N-acetylcysteine (NAC), and Mito-TEMPO were purchased from Sigma.

### Knockdown of LC3 by short harpin RNA (shRNA)

shRNA trasfection in A549 was carried out according to the manufacturer’s protocol using TransIT-X2 dynamic delivery system (Mirus Bio LLC). The sequence of the LC3 shRNA targets LC3 mRNA (NM_022818) is CGCTTACAGCTCAATGCTAAT and shRNA was obtained from the National Core Facility for Manipulation of Gene Function by RNAi, miRNA, miRNA sponges, and CRISPR / Genomic Research Center, Academia Sinica. The LC3 shRNA and scramble shRNA were transfected for 72 h following by Acr treatment as described above.

### Acr cytotoxicity by MTT and LDH assay

The cytotoxicity of Acr was determined using a modified 3-(4,5- dimethylthiazol-2-yl)-2,5-diphenyl tetrazolium (MTT; Sigma, St. Louis, MO) assay and a lactate dehydrogenase (LDH) leakage assay, as previously described [59]. All of these experiments were performed in triplicate and were repeated independently at least three times.

### Genomic isolation

Genomic DNA was isolated as previously described [7, 8, 26].

### Quantitative real-time RT-PCR

Total RNA isolation, reverse transcription and subsequent real-time RT-PCR analysis of cDNA were previously described [59]. The primers (5'-3') were: AACCCGTCATCTACTCTACCATCT and GCTTCTGTG GAA CGAGGGTTTATTT for ND2; TCACAACACCCTAGGCTCACTAA and GGGAGT CATAAGTGGAGTCCGT for ND4; CGTACGCCTAACCGCTAACATT and GCGACA GCGATTTCTAGGATAGT for ATPase 6; GCCCTTTTCCTAACACTCACAACAA and GTAAAGGATGCGTAGGGATGGG for CO2; CCGTCTAGAAAAACCTGCC and GCCAAATTCGTTGTCATACC for GAPDH. To calculate the relative RNA expression, GAPDH was used as an internal control for all qRT-PCR reactions and compared with control groups.

### Western blot analysis

A549 and MRC-5 cells were treated with Acr as indicated, then cell lysates were prepared and analyzed as previously described [59]. Briefly, blots were probed with a monoclonal antibody against ATG7 (1:1000, Cell signaling, #2631), caspase-3 (1:1000, Cell signaling, # 9662), caspase-9 (1:1000, Cell signaling, # 9502), Drp1 (1:1000, BD, # 611112), LC3 (1:1000, Cell signaling, # 2775), OPA1 (1:1000, BD, # 611112), PARP-1 (1:1000, Cell signaling, # 9542), Pink1 (1:1000, BD, # BC100-494), p-Drp1 (Ser616, 1:1000, Cell signaling, # 3455) and β-actin antibody (1:5,000; Millipore [clone C4]) at 4 °C for overnight following by horseradish peroxidase-conjugated secondary IgG (1:3,000; Millipore) for 1 h at room temperature. The immunoreaction was visualized using Enhanced Chemiluminescence (ECL) (Millipore Corporation, Billerica, MA).

### UvrABC and T4 endonuclease V incision assay

UvrABC and T4 incision assays were the same as described [60, 61]. Briefly, genomic DNA was extracted from Acr-treated cells and incubated with UvrABC or T4 endonuclease V (T4 endo. V) at 37°C for 1 h. After UvrABC or T4 endo. V incision, DNA was purified in the same manner as described above and dissolved in TE buffer. Incised DNA was denatured in 90% formamide at 45°C for 1 h and separated by electrophoresis in 0.6% of agarose gels. It is worth noting that UvrABC specifically incises bulky DNA damages, including Acr-induced PdG adducts [31, 32] and T4 endo. V incises UV-induced cyclobutane pyrimidine dimers (CPDs) [62].

### Immunofluorescence assay

Acr-treated or control cells on coverslips of 6-well chambers were incubated with MitoTracker Red CM-H2XRos (Invitrogen, M7513) or with MitoTracker Green FM (Invitrogen, M7514), or Lysotracker Red DND-99 (Invitrogen, L7528), as indicated by the manufacturer’s instructions. After staining, cells were washed with PBS followed by immunofluorescent staining procedures, as previously described [59]. The following antibodies were used at the noted dilutions: anti-LC3 antibody (1:100, Cell Signaling, # 2775), anti-PINK1 antibody (1:100, BD, # BC100-494), anti-Drp1 antibody (1:100, BD, # 611112) at 4 °C for overnight. Monodansylcadaverine (MDC) has been proposed as a tracer for autophagic vacuoles [63]. A549 and MRC-5 cells were cultured on coverslips overnight, treated with Acr for 8 h, rinsed with PBS, then stained with MDC (Sigma, 50 μM) and MitoTracker Red CM-H2XRos (Invitrogen, M7513), at 37°C for 30 min. The cells were fixed for 15 min with ice-cold 4% paraformaldehyde at 4°C, washed with PBS, and examined under Olympus FV1000 confocal laser microscopy. Morphology of mitochondrial was stained with MitoTracker Red CM-H2XRos (Invitrogen, M7513) described the above and quantified byMicroP3D software [64].

### Intracellular ROS detection

The pro-oxidants were measured as previously described [59]. Briefly, A549 or MRC-5 cells were treated with Acr (0-100 μM) or hydrogen peroxide (H_2_O_2_, 250 μM) as a positive control for 1 h, the culture medium was replaced with 2′7′- dihydrodichlorofluorescein diacetate (H2DCFDA, 5 μg/mL, Sigma) and levels of pro-oxidants were determined by flow cytometry (FACS Calibur, BD).

### MitoSox staining assay

A549 and MRC-5 cells were treated with Acr (0-100 μM) or hydrogen peroxide (H_2_O_2_, 250 μM) as positive controls at room temperature and then incubated with MitoSox red (5 μM, Molecular probe, M36008) for 30 min as indicated by the manufacturer’s instructions.

### Mitochondrial membrane potential detection

JC-1 was used to determine the mitochondrial membrane potential [65]. Briefly, A549 and MRC-5 cells were treated with Acr (0-100 μM) or carbonyl cyanide *m*-chlorophenyl hydrazone (CCCP, 100 μM, Sigma) for 1 h as a positive control. The cells were harvested and re-suspended in PBS containing JC-1 dye (Moleculare Probe, T3168) (5 μM) for 30 min at room temperature in the dark and fluorescence intensity levels were determined by flow cytometry (FACS Calibur, BD).

### ATP measurement

A549 and MRC-5 cells were treated with Acr (0-100 μM) or CCCP (100 μM, Sigma) for 1 h as a positive control. The cells were harvested, lysed using cell lysis reagent and the ATP content in the cell lysates was measured using the ATP bioluminescence assay kit HS II (Roche Molecular Biochemicals) with a Multimode microplate reader (TECAN, Infinite 200).

### Mitochondrial respiration analysis

Mitochondrial respiration was assessed using a Seahorse XF24 Extracellular Flux Analyzer (Seahorse Bioscience, North Billerica, MA) according to manufacturer’s instructions. Briefly, A549 and MRC-5 cells were cultured on Seahorse XF-24 plates at a density of 3x10^4^ cells/ well. The cells were treated with Acr (100 μM for A549 cells; 50 μM for MRC-5 cells) for 1 h. After the assays, plates were saved and protein readings were measured for each well to confirm equal cell numbers per well. Respiration rates were presented as the mean ± SEM of 4 independent experiments in all experiments performed with 4 to 10 replicate wells in the Seahorse XFe24 analyzer.

### Determination of mtDNA copy number

Total DNAs were extracted from Acr-treated cells and mtDNA copy number was determined as described previously [66].

### nDNA and mtDNA damage assay

Genomic DNA damages, including both nDNA and mtDNA damages, were assessed using a well-validated, sensitive Q-PCR assay that relies on the principle that oxidative DNA lesions inhibit DNA polymerases (e.g., *Taq* and *rTth* DNA polymerase) [67]. Briefly, genomic DNA was isolated from Acr-treated cells, as described above. PCR was performed using Phusin High fidelity DNA polymerase (Thermo Scientific) with specific primers to amplify short (<250-bp) and long fragments (8.9 kb) of the mtDNA and nDNA (13.5 kb for the *β-globin* gene). The number of mitochondrial lesions was calculated by the equation, D = (1–2^−(Δlong−Δshort)^) × 10,000 (bp)/ size of the long fragment (bp).

### Cell cycle analysis and propidium iodide (PI)/Annexin V-FITC analysis

Cell cycle analysis and PI/ Annexin V analysis were performed as previously described [59]. Briefly, for cell cycle analysis, Acr-treated cells were fixed in ice-cold 70% ethanol, digested with DNase-free RNase A (50 U/ ml) and stained with propidium iodide (PI, 10 μg/ ml; Sigma) followed by flow cytometry analysis. For PI/ Annexin V analysis, Acr-treated cells were analyzed by FITC Annexin V Apoptosis Detection Kit I (BD Biosciences Canada, Mississauga, ON, Canada).

### Statistical analyses

Student’s *t*-tests were used to determine statistical significance, and two-tailed *P*-values were shown. A minimum of three independent replicate experiments was performed to justify the use of these statistical tests.

## CONCLUSION

In summary, this study demonstrates that acrolein induces mitochondrial DNA damages, decreases mitochondrial membrane potential, reduces mitochondrial respiration, and ultimately triggers cellular apoptosis. The process is not reversed by autophagy or mitophagy. We propose that these effects induced by acrolein result in pulmonary epithelial injury and contribute to the etiology of lung diseases.

## SUPPLEMENTARY MATERIALS FIGURES


