# Supplementary material for: Acrolein induces mtDNA damages, mitochondrial fission and mitophagy in human lung cells
Source: Oncotarget. 2017 Jul 31;8(41):70406–21. doi: 10.18632/oncotarget.19710 (PMC5642564; doi:10.18632/oncotarget.19710)
Supplement: Supplementary file 1 [file oncotarget-08-70406-s001.pdf]

Acrolein induces mtDNA damages, mitochondrial fission and mitophagy in human lung cells

SUPPLEMENTARY MATERIALS

REFERENCES

1. Feng Z, Hu W, Hu Y, Tang MS. Acrolein is a major cigarette-related lung cancer agent: Preferential binding at p53 mutational hotspots and inhibition of DNA repair. Proc Natl Acad Sci U S A. 2006; 103:15404-9.
2. Tang MS. (1996). Technologies for Detection of DNA Damage and Mutation. ed Pfeifer G (Plenum: New York). pp. 139-52.
3. Vreeswijk MP, van Hoffen A, Westland BE, Vrieling H, van Zeeland AA, Mullenders LH. Analysis of repair of cyclobutane pyrimidine dimers and pyrimidine 6-4 pyrimidone photoproducts in transcriptionally active and inactive genes in Chinese hamster cells. J Biol Chem. 1994; 269:31858-63.
4. Lee HC, Yin PH, Yu TN, Chang YD, Hsu WC, Kao SY, Chi CW, Liu TY, Wei YH. Accumulation of mitochondrial DNA deletions in human oral tissues -- effects of betel quid chewing and oral cancer. Mutat Res. 2001; 493:67-74.

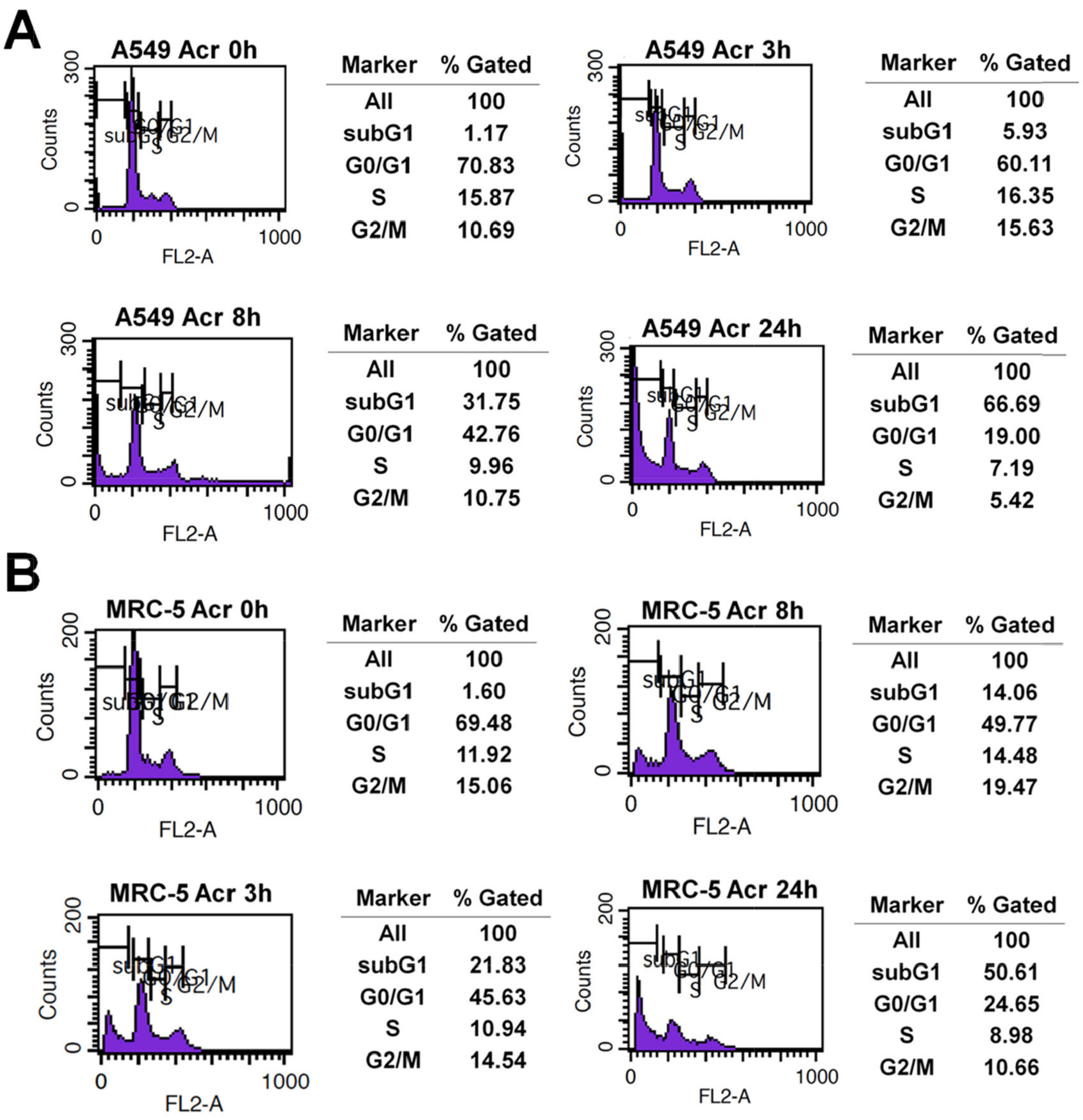

Supplementary Figure 1: Acr induces sub-G1 subpopulation using cell cycle analysis. In panels **(A & B)**, cells were treated with Acr (A549: 75 μM; MRC-5: 25 μM) for different times and cell cycle phases were determined by PI staining followed by flow cytometry analysis as described in materials and methods. The cell cycle characteristics were quantified as follows: sub-G1, G0/G1, S and G2/M phase.

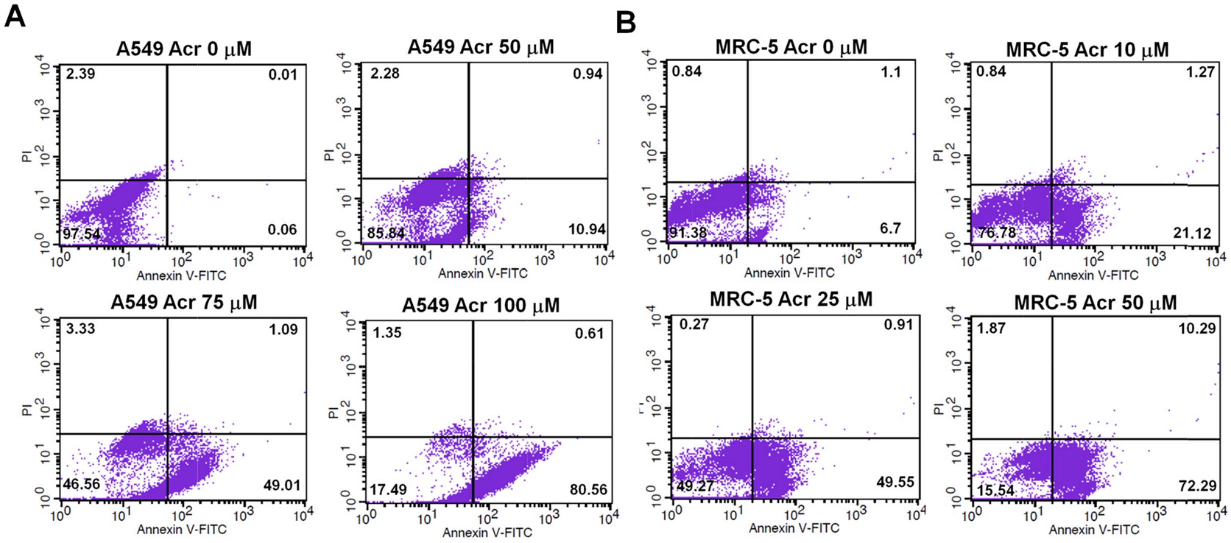

Supplementary Figure 2: Acr induces apoptosis using PI/ Annexin V-FITC analysis. In panels **(A & B)**, cells were treated with different concentrations of Acr (A549: 0-100 μM; MRC-5: 0-50 μM) for 24 h and analyzed by propidium iodide (PI)/ Annexin V-FITC staining followed by flow cytometry analysis as described in materials and methods. Annexin V-FITC fluorescence was detected on the FL-1 detector and PI fluorescence on the FL-2 detector. Four populations of cells were analyzed and quantified as percentage of total cells: live control cells (Annexin V−/PI−); early stage apoptotic cells (Annexin V+/PI−); late stage apoptotic cells (Annexin V+/PI+); necrotic cells (Annexin V−/PI+).

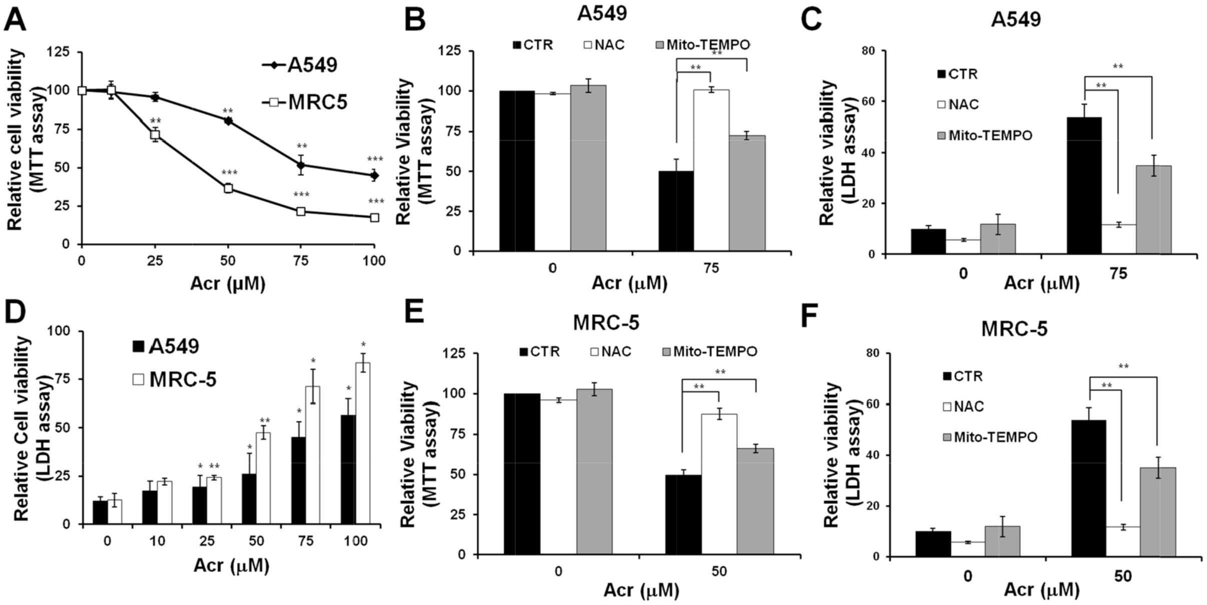

Supplementary Figure 3: Effect of NAC or Mito-Tempo on Acr-induced cytotoxicity in lung adenocarcinoma (A549) and normal human lung fibroblasts (MRC-5). Exponentially growing A549 cells and MRC-5 cells were treated with different concentrations of Acr for 24 h and the cell survival was determined by MTT assay (panel **A**) and LDH leakage assay (panel **D**). After cells were pre-treated with NAC (5 mM) or Mito-TEMPO (100 μM) for 1 h, Acr (A549: 75 μM; MRC-5: 25 μM) was added in cells for 24 h followed by MTT assay (panels **B** & **E**) and LDH leakage assay (panels **C & F**), as described in materials and methods.

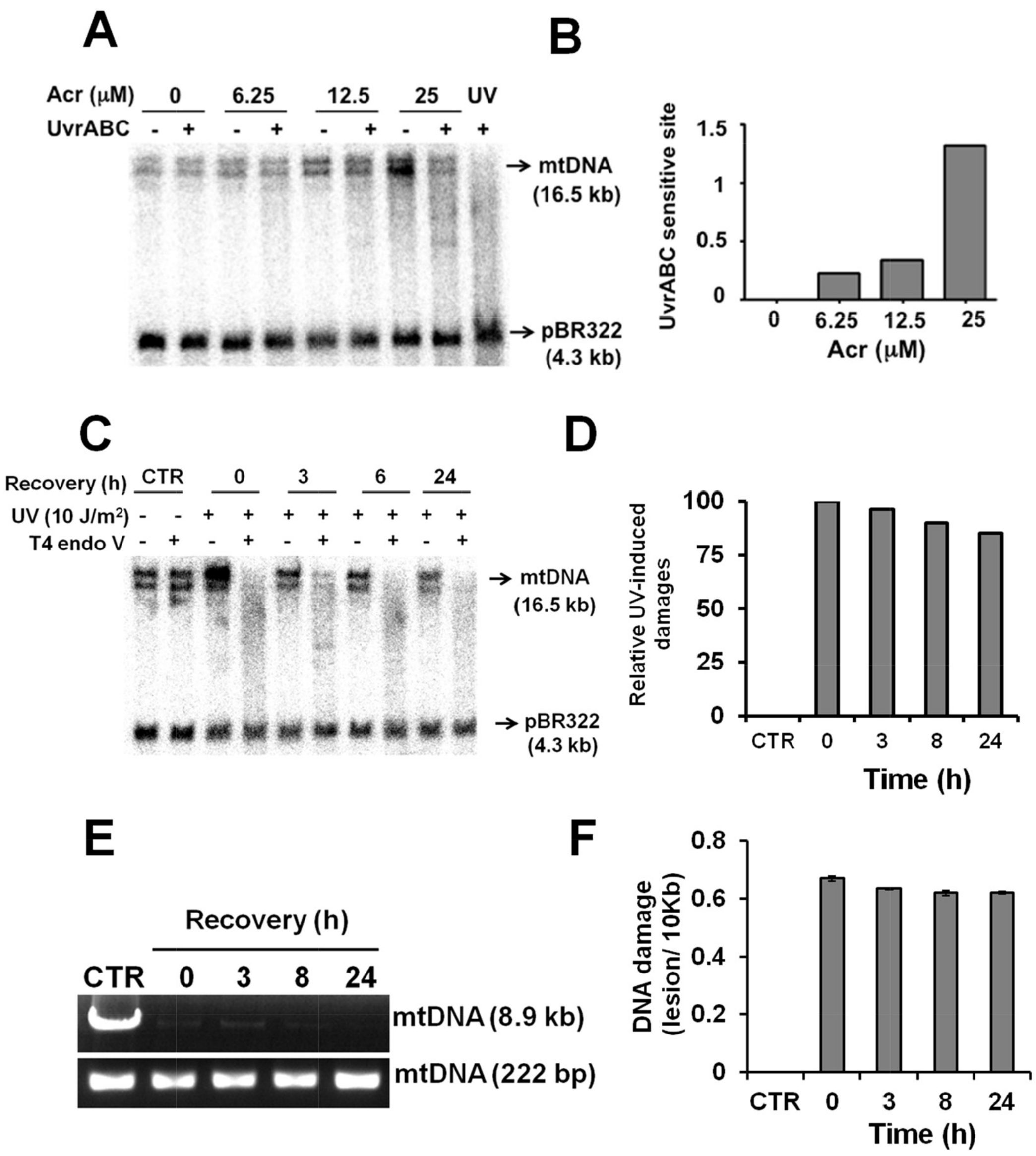

Supplementary Figure 4: Acr induces UvrABC-sensitive PdG adducts in mtDNA and mitochondria are deficient in nucleotide excision repair (NER). The UvrABC incision assay and the separation of the resultant DNA were carried out as previously described [1]. It is well established that the three UvrABC proteins work in concert to incise the UVC photoproducts CPD and pyrimidine < 6–4 > pyrimidone [2, 3]. In panel **(A)**, A549 cells were exposed to 6.25, 12.5, or 25 μM of Acr for 3 h, lysed, the high molecular weight DNA was extracted, and the mtDNA was linearized by BamHI digestion. The DNA was then reacted with (+) or without (-) UvrABC which incises bulky DNA damage specifically. The resultant DNAs were denatured with 95% formamide, separated by electrophoresis in a 0.5% agarose gel, transferred to nylon membrane, and hybridized with 32P-labled mtDNA and pBR322 probes. Linearized pBR322 was added to samples as input controls. Cells irradiated with UVC (10 J/m2) were used as positive control for UvrABC incision assay. In panel **(B)**, The UvrABC sensitive sites in mtDNA were quantified, as previously described [1]. In panel **(C)**, A549 cells were UV irradiated, then incubated in growth medium for different periods of time (0, 3, 8, and 24 h) at 37 ^{o}C. At the end of incubation, T4 endonuclease V (T4 endo. V) sensitive sites in mtDNA were analyzed as panel A. In panel **(D)**, relative UV-induced damages in mtDNA were calculated using T4 endo. V sensitive sites, as described in panel B. Note: mtDNAs remain sensitive to T4 endo. V cut, indicating that UV-induced photoproducts are not repaired. In panel **(E)**, after A549 cells were treated with Acr (100 μM, 6 h), cells were washed with PBS and incubated in growth medium for different periods of time (0, 3, 8, and 24 h) at 37 ^{o}C. DNA damages in mtDNA were analyzed using QPCR with specific primers against mtDNA (8.9 kb, 222 bp) and panel **(F)** shows quantifications of Acr-induced mtDNA damages in cells, as described in materials and methods. Bar graphs are of data collected from 3 independent experiments. Data are mean ± s.d.

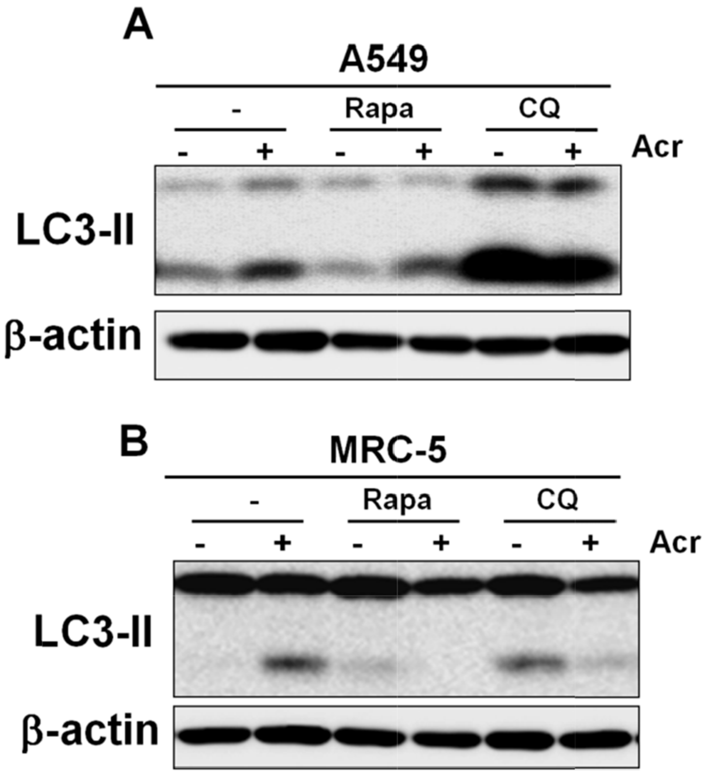

Supplementary Figure 5: Effect of autophagy inhibitors or inducers on Acr-induced autophagy pathway. In panels **(A & B)**, A549 or MRC-5 cells were pretreated with autophagy inhibitors, chloroquine (CQ, 20 µM), or with the autophagy inducer rapamycin (Rapa,100 µM) for 1 h, then co-treated with Acr (A549: 100 µM; MRC-5: 50 µM) for 6 h at 37 °C. Cleavage of LC3-II was analyzed using western blot analysis with anti-LC3 antibody.

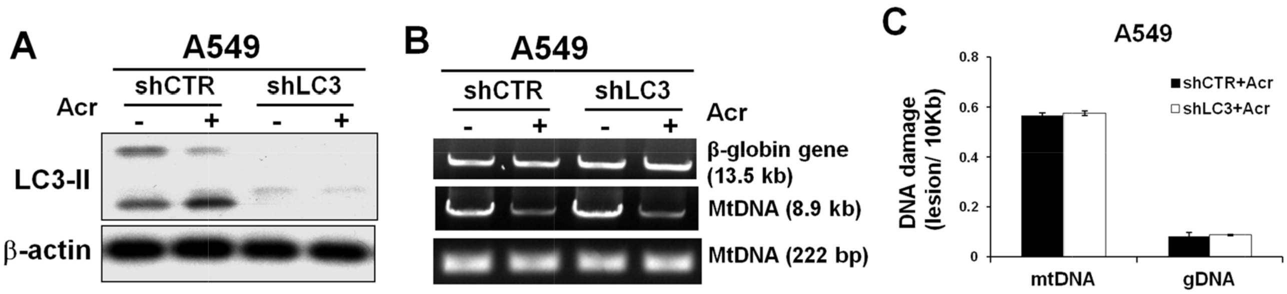

Supplementary Figure 6: Effect of LC3 shRNA knockdown on Acr-induced DNA damages. A549 cells were transfected with LC3 shRNA plasmid for 72 h, and then treated with Acr (100 µM) for 6 h at 37 ^{o}C. In panel **(A)**, cleavage of LC3-II was analyzed using western blot analysis. In panels **(B & C)**, mtDNA and nDNA damages were analyzed, as described in materials and methods (panel B), and quantified as shown (panel C).

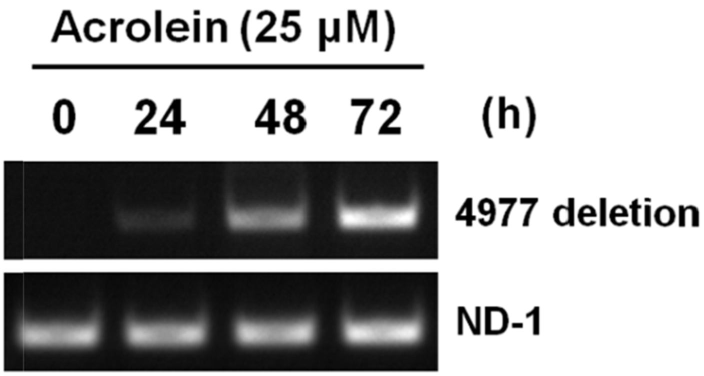

Supplementary Figure 7: Acr induces 4977 deletion in mtDNA in A549 cells. After A549 cells were treated with Acr (25 µM for 24-72 h), DNA was extracted and analyzed using semi-quantitative PCR with specific primers against ND1 as total mtDNA and the amplification of a 524 bp PCR product from the 4977 bp deleted mtDNA as previously described [4].
